# Predicting postoperative recurrence and survival in malignant ovarian germ cell tumors: a single-center retrospective cohort study and nomogram development

**DOI:** 10.3389/fonc.2026.1768796

**Published:** 2026-04-07

**Authors:** Yun Feng, Zhiyu Feng, Ruixia Guo

**Affiliations:** 1 Department of Gynaecology, The First Affiliated Hospital of Zhengzhou University, Zhengzhou, Henan, China; 2 Qiqihar Medical University, Qiqihar, Heilongjiang, China

**Keywords:** carcinoembryonic antigen (CEA), malignant ovarian germ cell tumors (MOGCTs), nomogram, overall survival, prognostic model, recurrence, risk factors

## Abstract

**Background:**

Malignant ovarian germ cell tumors (MOGCTs) are associated with a relatively good prognosis when diagnosed early and treated appropriately. However, disease recurrence significantly worsens outcomes. Therefore, identifying risk factors for recurrence is crucial for improving patient prognosis. The role of carcinoembryonic antigen (CEA) in MOGCTs remains unclear, and this study aimed to evaluate its prognostic value.

**Methods:**

This single-center retrospective study included 123 MOGCT patients treated between January 2012 and December 2024. Data on clinical characteristics, serological markers (including CEA), surgical details, and follow-up outcomes were collected. Cox proportional hazards regression analyzed risk factors for postoperative recurrence and all-cause mortality. Kaplan-Meier curves evaluated progression-free survival (PFS) and overall survival (OS). Nomograms were developed to predict the probabilities of recurrence and mortality.

**Results:**

Multivariate analysis identified that advanced International Federation of Gynecology and Obstetrics (FIGO) stage (II–IV) (HR = 5.299, P = 0.004), presence of metastasis (HR = 3.364, P = 0.015), and elevated preoperative CEA (HR = 4.237, P = 0.019) as independent risk factors for postoperative recurrence; For mortality, stage II–IV disease (HR = 5.398, P = 0.044) and elevated preoperative CEA (HR = 5.779, P = 0.012) were identified as risk factors. Predictive nomograms for recurrence and OS were successfully established.

**Conclusions:**

Tumor stage, metastasis status, and preoperative CEA level are predictive of postoperative recurrence in MOGCTs, while tumor stage and preoperative CEA level also predict postoperative OS. These findings may facilitate the identification of high-risk patients for early intervention. Further research is needed to explore the relationship between dynamic changes in CEA and the risk of recurrence. Although the routine application of CEA in MOGCTs requires more evidence, this study suggests it may serve as a potential supplementary prognostic indicator.

## 1 Introduction

Germ cell tumors (GCTs) are malignant tumors originating from primordial germ cells (i.e., the precursor cells that form germ cells during embryonic development). These tumors can occur within the gonads (such as the ovaries and testes) or outside the gonads (such as the mediastinum, retroperitoneum, and sacrococcygeal region) (1). GCTs are usually highly invasive and have the potential to metastasize. Comprehensive treatment methods such as surgery, chemotherapy, and radiotherapy are often used to improve the survival rate of patients. Among them, malignant ovarian germ cell tumors (MOGCTs) are the second most common type of ovarian malignant tumors, accounting for about 15 - 20% of all ovarian tumors (2). In the field of gynecological oncology, MOGCTs occupy an important position, especially in young women (especially adolescents and those under 40 years old), with the peak age of onset being 15–19 years old. Notably, MOGCTs account for about 70% of ovarian malignant tumors in this age group (3–5).

Overall, the prognosis of MOGCTs is relatively good. Most patients can achieve a high cure rate and good prognosis after being diagnosed at an early stage and receiving standardized treatment, and most patients can preserve their fertility (6). However, although surgery combined with chemotherapy can achieve complete remission in most MOGCT patients, a small number of patients will still experience persistent or recurrent diseases (7). For patients with persistent, refractory, or platinum-resistant recurrent diseases, their prognosis deteriorates significantly (8). Therefore, identifying the risk factors that influence recurrence and overall survival in MOGCTs is of great significance for improving patient prognosis.

Currently, most population-based studies focus on children and adolescents (9). However, there is still a relative lack of systematic research on the diagnosis and treatment patterns and prognosis of MOGCTs as a specific histological type in women of all ages, especially regarding the risk factors for recurrence and overall survival across all age groups in women. Furthermore, while markers such as alpha-fetoprotein (AFP) and beta-human chorionic gonadotropin (β-hCG) are utilized in the management of MOGCTs, the role of carcinoembryonic antigen (CEA) has not been clearly established, and its prognostic value is controversial. To fill this knowledge gap and explore the prognostic significance of various preoperative serum markers, including CEA, this study aims to explore the risk factors influencing recurrence and overall survival in MOGCTs and to construct a predictive model to help clinicians better assess patient prognosis.

## 2 Methods

### 2.1 Study design and population

This was a single-center, retrospective cohort study. Patients diagnosed with MOGCT who were eligible were recruited at the First Affiliated Hospital of Zhengzhou University between January 2012 and December 2024. The inclusion criteria were as follows (1): histologically confirmed MOGCT (2); no history of infertility or other malignancies; and (3) completeness of clinical data and follow up. Patients who did not meet the inclusion criteria were excluded from the study. This study was approved by the Ethics Committee of the First Affiliated Hospital of Zhengzhou University (approval number: 2025-KY-0286). As a retrospective study, the requirement for informed consent was waived by the ethics committee, and all data were anonymized.

### 2.2 Data collection and definitions

Clinical data, including information on age at diagnosis, previous medical history, histological type, International Federation of Gynecology and Obstetrics (FIGO) stage, pre-operative serum markers (AFP, CA125, CA199, CEA, HE4, β-hCG levels), tumor size, surgical details (open/laparoscopic, fertility-sparing surgery), and postoperative adjuvant chemotherapy regimens (if applicable) were obtained from detailed medical records. Survival and reproductive outcomes were assessed and recorded during the follow-up visit. All pathological specimens were reported by gynecological pathologists at the medical institutions. Histological types of tumors were classified according to the World Health Organization 2020 criteria. Recurrence was defined as the occurrence of local recurrence or distant metastasis (including lymph node, lung, and bone metastases) confirmed by imaging (CT/MRI) or pathology after surgical resection. The progression-free survival (PFS) was used as the study outcome, which refers to the time from the date of surgical resection to the occurrence of recurrence or distant metastasis (including lymph node, lung, and bone metastases, etc.) after surgery. Overall Survival (OS)is defined as the time period from the commencement of surgery to the death of the patient due to all causes. Follow-up was conducted via outpatient review or telephone until December 31, 2024. The median follow-up time was 58 months (range: 6–152 months). Serum markers were considered “elevated” if they exceeded the upper limit of the normal reference range provided by our hospital laboratory (see **Table 1** footnotes). For items not tested, they were treated as “missing” in the analysis.

**Table 1:** Characteristics of patients (n = 123).

Characteristics		
Ages, years	Mean ± SD	22.67 ± 12.08
	≤22.67 years, n(%)	68 (55.28)
	>22.67 years, n(%)	55 (44.72)
Parity, n (%)	Nulliparous	85 (69.11)
	Parous	38 (30.89)
Previous history of surgery, n (%)	No	83 (67.48)
	Yes	40 (32.52)
Preoperative serum AFP level^a^, n (%)	Not elevated	35 (28.46)
	Elevated	85 (69.10)
	Not checked	3 (2.44)
Preoperative serum β-HCG level^b^, n (%)	Not elevated	53 (43.09)
	Elevated	27 (21.95)
	Not checked	43 (34.96)
Preoperative serum CEA level^c^, n (%)	Not elevated	99(80.49)
	Elevated	24(19.51)
Preoperative serum CA125 level^d^, n (%)	Not elevated	31 (25.20)
	Elevated	88 (71.54)
	Not checked	4 (3.25)
Preoperative serum CA199 level^e^, n (%)	Not elevated	84 (68.29)
	Elevated	33 (26.83)
	Not checked	6 (4.88)
Preoperative serum HE4 level^f^, n (%)	Not elevated	58 (47.15)
	Elevated	26 (21.14)
	Not checked	39 (31.71)
Peritoneal cytology, n (%)	Negative	115 (93.50)
	Positive	8 (6.50)
Surgery mode, n (%)	Open	73 (59.35)
	Laparoscopy	50 (40.65)
Tumor size, cm (%)	Mean ± SD	15.94 ± 7.83
	≤16	73 (59.35)
	>16	50 (40.65)
Tumor rupture, n (%)	No	70 (56.91)
	Yes	53 (43.09)
Histologic type, n (%)	Yolk sac tumor	39 (31.71)
	Immature teratoma	35 (28.46)
	Mixed	23 (18.70)
	Dysgerminoma	21 (17.07)
	Choriocarcinoma	5(4.07)
FIGO stage, n (%)	I	77 (62.60)
	II	22 (17.89)
	III	21 (17.07)
	IV	3 (2.44)
Metastasis, n (%)	No	99 (80.49)
	Yes	24 (19.51)
Lymph node metastasis, n (%)	No	27 (21.95)
	Yes	4 (3.25)
	Not sampled	92 (74.80)
Residual tumor, n (%)	No	116 (94.31)
	Yes	7 (5.69)

CA125, cancer antigen 125; CA199, cancer antigen 199; AFP, alpha-fetoprotein; β-hCG, beta-human chorionic gonadotropin; CEA, carcinoembryonic antigen; FIGO, the international Federation of Obstetrics and Gynecology; HE4, human epididymis secretory protein 4.

a Reference range: 0–7 ng/mL.

b Reference range: 0–5 IU/L.

c Reference range: 0–5 ng/mL.

d Reference range: 0–35 U/mL.

e Reference range: 0–37 U/mL.

f Reference range: 0–90 pmol/L.

### 2.3 Perioperative treatment

Surgical approaches included open and laparoscopic surgery, with fertility-sparing surgery performed whenever possible for patients desiring future fertility. Postoperative adjuvant chemotherapy was decided based on FIGO stage, pathological type, and high-risk factors, primarily utilizing platinum-based regimens (e.g., BEP). Treatment decisions were made by a multidisciplinary team (MDT) according to contemporary clinical guidelines and individual patient circumstances.

### 2.4 Statistical analysis

The SPSS 26.0 software and R 4.3.1 were used for statistical analysis of the data. Count data were presented as cases (%). After data preprocessing, statistical analysis was performed. Continuous variables (e.g., age, CEA) were dichotomized for analysis using the mean value or clinically accepted cut-off points (e.g., CEA: 5 ng/mL). The Cox proportional hazards regression model was used to analyze the risk factors for postoperative recurrence s in patients with germ cell tumors. The Kaplan-Meier method was used to estimate progression-free survival (PFS) and overall survival (OS), and differences between groups were compared using the Log-rank test. The analysis was based on the results of the multivariate Cox proportional hazards regression model. Based on the independent prognostic factors identified in the multivariate Cox analysis, nomogram models for predicting PFS and OS were constructed using R software. All statistical tests were two-sided, and P < 0.05 was considered statistically significant.

## 3 Results

### 3.1 Clinicopathological characteristics and outcome events

During the study period, 123 patients with MOGCT underwent surgery at the First Affiliated Hospital of Zhengzhou University. **Table 1** lists the characteristics of the 123 patients. The mean age (± SD) of the patients was 22.67 years (± 12.08 years), and 85 patients (69.11%) were nulliparous. 40 patients (32.52%) had a history of previous surgery.

Serological examinations revealed that 85 patients (69.10%) had elevated AFP levels, 27 (21.95%) had elevated β-HCG levels, 24 (19.51%) had elevated CEA levels, 88 (71.54%) had elevated CA125 levels, 33 (26.83%) had elevated CA199 levels, and 26 (21.14%) had elevated HE4 levels. Moreover, pre-operative examinations showed that 115 patients (93.50%) had negative ascites cytology, while 8 patients (6.5%) had positive ascites cytology.

Regarding surgical approach, 73 patients (59.35%) underwent open surgery, and 50 (40.65%) underwent laparoscopic surgery. Seventy-seven patients (62.60%) underwent fertility-sparing surgery. The mean size (± SD) of the tumor was 15.94cm (± 7.83 cm). 53 patients (43.09%) had tumor rupture before and during surgery. Yolk sac tumor was the most common histological type (39 cases, 31.74%), followed by immature teratoma, mixed MOGCT, dysgerminoma, and choriocarcinoma. Seventy-seven patients (62.60%) had FIGO stage I disease, 22 (17.89%) had stage II, 21 (17.07%) had stage III, and 3 (2.44%) had stage IV. 24 patients (19.51%) had systemic metastasis, while 4 patients (3.25%) were diagnosed with lymph node metastasis. Seven patients (5.69%) still had residual tumors after surgery. A total of 119 patients (96.75%) received postoperative adjuvant chemotherapy, of whom 104 (87.40%) received platinum-based regimens. During the entire follow-up period, 22 recurrence events (17.9%) and 10 death events (8.1%) were observed.

### 3.2 Univariate and multivariate Cox regression analyses for identifying the risk factors for recurrence

Univariate analysis revealed that patient age, peritoneal cytology results, tumor stage, metastasis status, CEA level, and CA199 level were associated with postoperative recurrence (all P<0.1, **Table 2**). Multivariate analysis (including variables with P<0.1 from univariate analysis) showed that tumor stage, metastasis status, and preoperative CEA level were independent risk factors for postoperative recurrence (P<0.05, **Table 2**). Specifically, compared with patients at FIGO stage I, those at stages II-IV had a hazard ratio of 5.299 (95% CI: 1.687-16.643, P = 0.004). Patients with tumor metastasis had a hazard ratio of 3.364 (95% CI: 1.268-8.922, P = 0.015). Patients with elevated preoperative CEA levels had a hazard ratio of 4.237 (95% CI: 1.274-14.095, P = 0.019).

**Table 2:** Univariate and multivariate Cox regression analyses of postoperative recurrence in MOGCTs.

Variables	Univariate analysis			Multivariate analysis		
	HR	95%CI	P value	HR	95%CI	P value
Age^a^ (>22.67 vs ≤22.67)	0.352	0.136-0.907	*0.031*	0.432	0.157-1.193	0.105
Tumor rupture (Yes vs No)	1.198	0.509-2.822	0.679			
Preoperative serum AFP level (Elevated vs Not Elevated)	2.040	0.800-5.197	0.135			
Preoperative serum CEA level (Elevated vs Not Elevated)	12.118	4.867-30.169	*0.000*	4.237	1.274-14.095	*0.019*
Preoperative serum CA125 level (Elevated vs Not Elevated)	1.567	0.622-3.948	0.341			
Preoperative serum CA199 level (Elevated vs Not Elevated)	1.944	1.030-3.669	*0.040*	1.348	0.451-4.033	0.593
Preoperative serum β-hCG level (Elevated vs Not Elevated)	0.739	0.232-2.358	0.610			
Preoperative serum HE 4 level (Elevated vs Not Elevated)	0.484	0.163-1.441	0.192			
Peritoneal cytology (Positive or Negative)	2.924	0.860-9.940	*0.086*	1.752	0.406-7.556	0.481
Metastasis (Yes vs No)	9.592	3.955-23.261	*0.000*	3.364	1.268-8.922	*0.015*
FIGO stage (I vs II-IV)	13.825	5.041-37.911	*0.000*	5.299	1.687-16.643	*0.004*
Surgery mode (Open vs Laparoscopy)	1.121	0.472-2.660	0.796			

A total of 22 recurrence events were observed. ^a^ divided by the mean value.

### 3.3 Analysis of independent risk factors for postoperative recurrence

**Figure 1:**
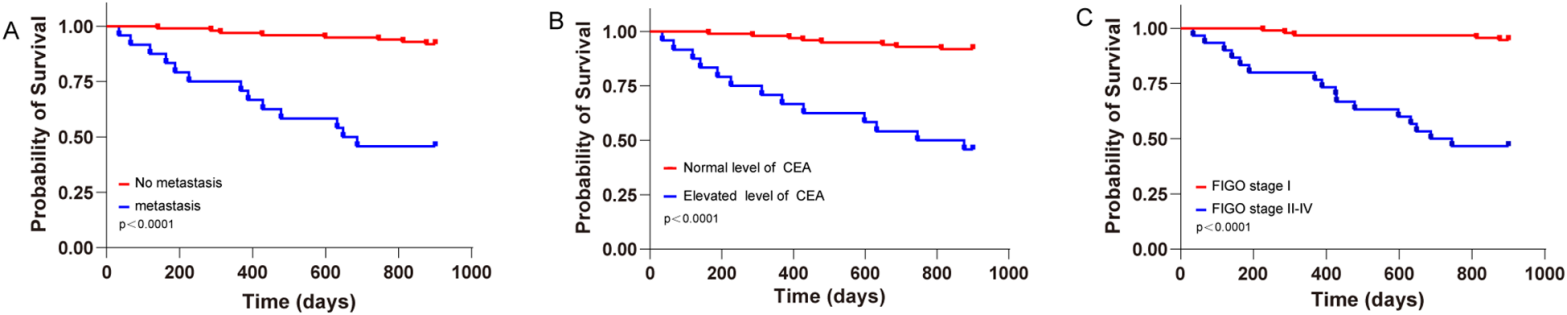
Kaplan-Meier curves for progression-free survival (PFS). Kaplan-Meier survival curves depicting the PFS of patients with MOGCTs based on different clinical parameters. **(A)** PFS comparison between patients with and without metastasis (p < 0.0001). **(B)** PFS comparison between patients with normal and elevated preoperative CEA levels (p < 0.0001). **(C)** PFS comparison between patients at FIGO stage I and those at stages II-IV (p < 0.0001).

For the three independent risk factors affecting postoperative recurrence in patients, the Kaplan - Meier method was used to plot the PFS curves (**Figures 1A–C**). The results showed that the postoperative progression - free survival rates of patients with FIGO stage I, those without metastasis, and those with preoperative serum CEA < 5 ng/mL were significantly higher than those of patients with stage II or above, those with metastasis, and those with CEA ≥ 5 ng/mL. The differences were statistically significant (all P < 0.05).

**Figure 2:**
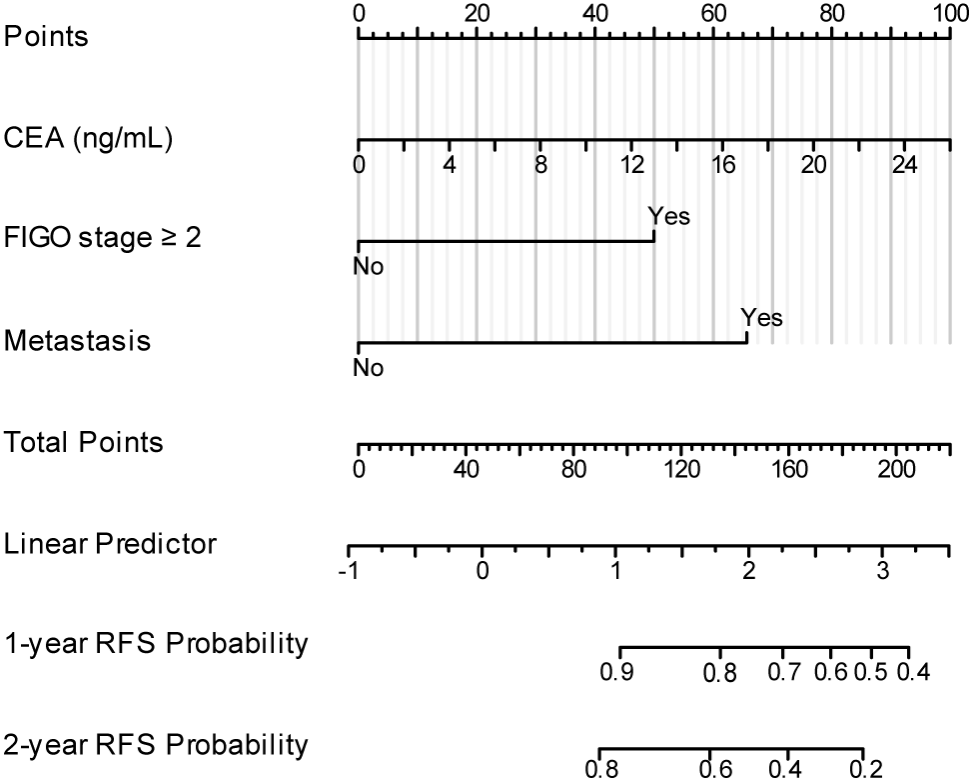
Nomogram for predicting 1-year and 2-year recurrence-free survival (RFS) in patients with MOGCTs. Instructions: For each predictor (Preoperative CEA, FIGO stage ≥II, Metastasis), locate the patient’s value on the corresponding axis, draw a line straight upwards to the ‘Points’ axis to determine the points for that predictor. Sum the points for all three predictors. Locate the total points on the ‘Total Points’ axis, then draw a line straight down to the bottom axes to read the predicted 1-year and 2-year RFS probabilities.

The nomogram model for predicting recurrence in patients with MOGCT was constructed based on influencing factors (**Figure 2**). To use the nomogram, locate the patient’s value for each predictor (CEA, FIGO stage, Metastasis) on the corresponding axis, draw a vertical line to the ‘Points’ axis to obtain the score for that predictor, sum all scores, and locate the total score on the ‘Total Points’ axis. Then, draw a vertical line down to the survival probability axes to read the predicted 1-year and 2-year recurrence-free survival probabilities. The nomogram for predicting recurrence demonstrated good discriminatory ability, with a Harrell’s Concordance Index (C-index) of 0.85 (95% CI: 0.78-0.92).

### 3.4 Univariate and multivariate Cox regression analyses for identifying the risk factors for mortality

Univariate analysis showed that preoperative CEA level, CA199 level, metastasis status, and FIGO stage were associated with OS (all P<0.1, **Table 3**). Multivariate analysis (considering factors with P<0.05 from univariate analysis) identified preoperative serum CEA level (HR = 5.779, P = 0.012) and FIGO stage (HR = 5.398, P = 0.044) as independent predictors of OS (**Table 3**).

**Table 3:** Univariate and multivariate Cox regression analyses of Overall Survival in MOGCTs.

Variables	Univariate analysis			Multivariate analysis		
	HR	95%CI	P value	HR	95%CI	P value
Age^a^ (>22.67 vs ≤22.67)	0.718	0.249-2.07	0.540			
Tumor rupture (Yes vs No)	0.977	0.339-2.816	0.966			
Preoperative serum AFP level (Elevated vs Not Elevated)	5.324	0.696-40.703	0.107			
Preoperative serum CEA level (Elevated vs Not Elevated)	12.789	4.002-40.868	*0.000*	5.779	1.481-22.558	*0.012*
Preoperative serum CA125 level (Elevated vs Not Elevated)	1.351	0.377-4.844	0.644			
Preoperative serum CA199 level (Elevated vs Not Elevated)	5.040	1.689-15.045	*0.004*	1.561	0.452-5.384	0.481
Preoperative serum β-hCG level (Elevated vs Not Elevated)	0.000	–	0.999			
Preoperative serum HE 4 level (Elevated vs Not Elevated)	2.902	0.779-10.809	0.112			
Peritoneal cytology (Positive or Negative)	1.096	0.143-8.382	0.929			
Metastasis (Yes vs No)	11.491	2.57-51.38	*0.001*	0.899	0.295-2.738	0.852
FIGO stage (I vs II-IV)	3.340	1.158-9.634	*0.026*	5.398	1.043-27.955	*0.044*
Surgery mode (Open vs Laparoscopy)	0.375	0.105-1.345	0.132			

A total of 10 death events were observed. ^a^ divided by the mean value.

### 3.5 Analysis of independent risk factors for OS

**Figure 3:**
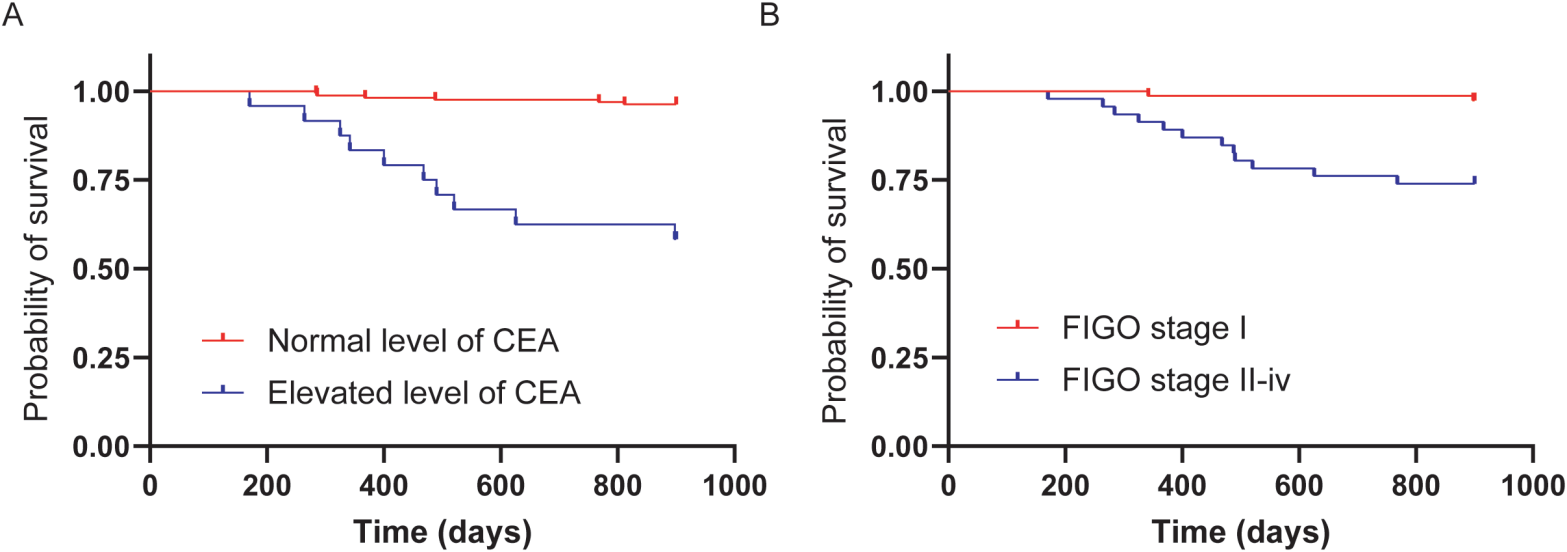
Kaplan-Meier curves for overall survival (OS). Kaplan-Meier survival curves depicting the OS of patients with MOGCTs based on different clinical parameters. **(A)** OS comparison between patients with normal and elevated preoperative CEA levels (p < 0.0001). **(B)** OS comparison between patients at FIGO stage I and those at stages II-IV (p < 0.0001).

For the assessment of OS in patients with MOGCTs, the Kaplan-Meier method was employed to generate survival curves based on distinct clinical parameters (**Figure 3**). Panel A of the figure illustrates the OS curves for patients categorized by normal versus elevated levels of CEA. Patients with normal CEA levels exhibited significantly higher OS rates compared to those with elevated CEA levels, with a statistically significant difference(p<0.0001). Panel B displays the OS curves for patients stratified by FIGO stage, contrasting those at stage I with those at stages II-IV. The results indicate a marked disparity in OS, with patients at stage I experiencing significantly better survival outcomes than those at more advanced stages (p<0.0001).

These findings underscore the impact of CEA levels and FIGO stage on the OS of MOGCT patients, highlighting their prognostic value. The Kaplan-Meier survival analysis confirms the clinical relevance of these factors, providing a clear visualization of the survival disparities among different patient groups. The statistically significant differences in survival probabilities suggest that both CEA levels and FIGO stage are critical in determining patient outcomes and should be considered in risk stratification and treatment planning for MOGCTs.

**Figure 4:**
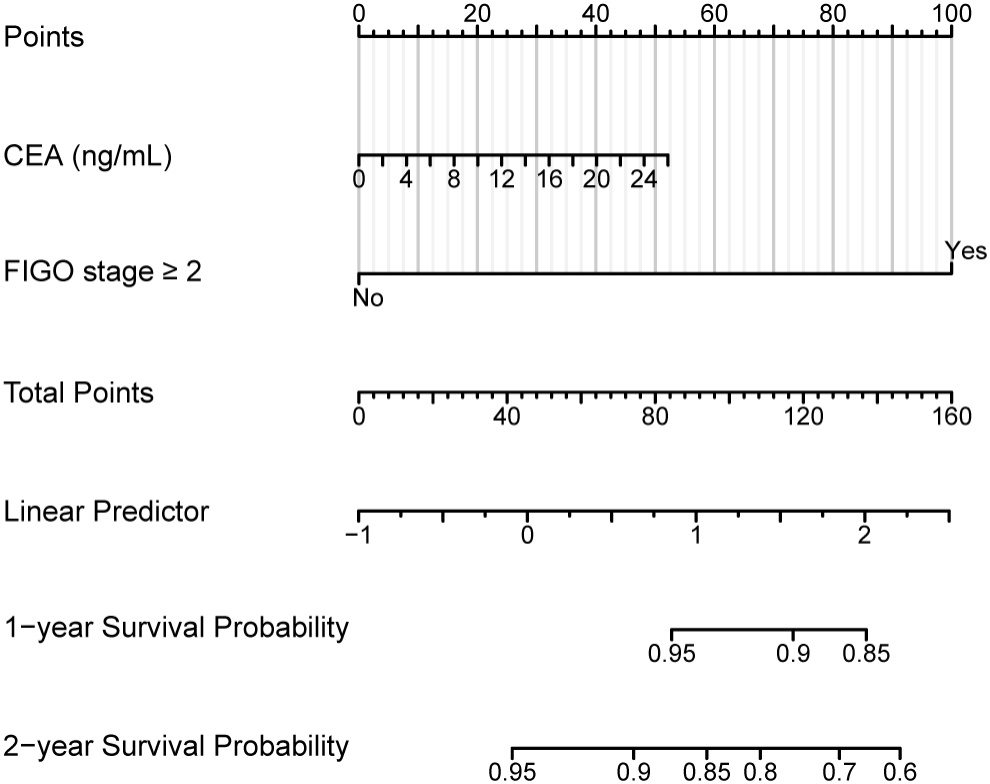
Nomogram for predicting 1-year and 2-year overall survival **(OS)** in patients with MOGCTs. Instructions: For each predictor (Preoperative CEA, FIGO stage ≥II), locate the patient’s value on the corresponding axis, draw a line straight upwards to the ‘Points’ axis to determine the points for that predictor. Sum the points for both predictors. Locate the total points on the ‘Total Points’ axis, then draw a line straight down to the bottom axes to read the predicted 1-year and 2-year OS probabilities.

**Figure 4** presents the Nomogram constructed for predicting the 1-year and 2-year survival probabilities of patients with MOGCTs. To use this nomogram, follow the same steps as described for **Figure 2**: locate the patient’s value for each predictor (CEA, FIGO stage) on the corresponding axis, draw a line to the ‘Points’ axis, sum the points, locate the total on the ‘Total Points’ axis, and draw a line down to the survival probability axes to read the predicted 1-year and 2-year overall survival probabilities. The nomogram for predicting OS also showed good discriminatory ability, with a C-index of 0.82 (95% CI: 0.74-0.90).

## 4 Discussion

### 4.1 Principal findings

Our study identified independent risk factors for postoperative recurrence and OS in patients with MOGCTs through univariate and multivariate Cox regression analyses. Specifically, tumor stage, the presence of tumor metastasis, and preoperative CEA levels were identified as independent risk factors for postoperative recurrence, while tumor stage and preoperative CEA levels were identified as independent risk factors for postoperative overall survival. Notably, elevated preoperative CEA demonstrated significant prognostic value, which has not been sufficiently addressed in previous literature on MOGCTs. Additionally, we constructed a nomogram to assist clinicians in more accurately predicting the risk of relevant events, thereby enabling the development of more personalized treatment strategies and closer follow-up for high-risk patients.

### 4.2 Comparison with existing literature and discussion on the role of CEA

Research on risk factors for recurrence and overall survival in MOGCTs is relatively limited. Consistent with established literature (10, 13, 14), our study confirmed that advanced FIGO stage is a strong, independent predictor of both recurrence and OS. In contrast, the effects of surgical approaches and chemotherapy regimens appear to be less definitive (10–12). This underscores the need for more robust and novel prognostic biomarkers to improve risk stratification.

Our study addresses this need by identifying preoperative serum CEA level as an equally crucial and independent prognostic factor, a finding that introduces a new perspective into the MOGCT prognostic landscape. CEA is a glycoprotein elevated in various carcinomas (15), with established utility in colorectal cancer surveillance (16). However, its role in MOGCTs remains ambiguous and underexplored, particularly regarding its baseline prognostic value.

Previous studies mainly explored the relationship between CEA and different histological types of MOGCTs (17), or as shown in the study by Talerman, A et al., it was considered that there was no correlation between serum CEA levels and disease activity (18) (this conclusion was based on the results of continuous CEA detection in 70 germ cell tumor patients). The apparent discrepancy between our findings and those of Talerman et al. may be reconciled by a key methodological distinction. Their study evaluated the utility of serial CEA for detecting recurrence during follow-up, whereas our analysis assessed the prognostic power of a single preoperative baseline CEA level. We posit that the preoperative CEA level may reflect the inherent biological aggressiveness or a specific differentiation state of the tumor at diagnosis, which is a distinct biological and clinical question from its role as a dynamic surveillance marker. This hypothesis is supported by the significant weight CEA carries in our recurrence-prediction nomogram, suggesting its unique value in initial risk assessment.

The prognostic exploration of other serum markers in MOGCTs, such as AFP, β-hCG, and CA125, has primarily focused on OS or shown inconsistent results for recurrence (19–23). Our work contributes by systematically evaluating multiple preoperative markers and identifying CEA as a strong, independent predictor specifically for recurrence—a critical endpoint directly impacting survival and clinical management.

### 4.3 Strengths, limitations, and clinical implications

The strengths of this study are notable given the context of MOGCTs. First, with a cohort of 123 patients, this represents one of the larger single-center studies on this rare tumor entity (accounting for <5% of all malignant ovarian tumors) (4), for which prospective trials are challenging. Second, it supplements the evolving evidence on the prognostic role of preoperative serum tumor markers, specifically highlighting CEA as a novel independent predictor for both recurrence and OS. Third, the developed nomograms provide an intuitive tool for individualized risk assessment.

Several limitations must be acknowledged. First, the retrospective, single-center design carries inherent risks of bias from unmeasured confounders and temporal changes in treatment strategies. Second, as the prognostic link with CEA was not prespecified, postoperative and dynamic CEA data were not systematically collected, precluding evaluation of CEA as a monitoring biomarker. Third, incomplete lymph node dissection in some patients may have led to staging inaccuracies. Finally, although the nomograms demonstrated good discriminatory ability in our cohort (C-index: 0.85 for recurrence, 0.82 for OS), the lack of an external validation cohort means their generalizability requires confirmation in independent populations.

Clinical Implications: Despite these limitations, our findings have implications for clinical practice. First, preoperative CEA testing is low-cost and widely available. Incorporating it into the preoperative assessment panel for MOGCTs may help further stratify risk beyond traditional factors, particularly for identifying potential high-recurrence-risk subgroups among FIGO stage I patients. Second, risk stratification based on the nomogram could guide individualized postoperative management. For high-risk patients (e.g., FIGO II-IV stage with elevated CEA), more intensive follow-up (e.g., shorter intervals for imaging), more proactive discussions on adjuvant therapy, or exploration of novel maintenance strategies could be considered. For low-risk patients, overtreatment and unnecessary examinations could be avoided, reducing patient burden. Of course, any adjustment to treatment strategies should be made within a multidisciplinary team (MDT) discussion, considering the patient’s specific situation.

### 4.4 Future research directions

Future multicenter, prospective studies are needed to validate the prognostic value of CEA in MOGCTs and explore the relationship between its dynamic changes and treatment response and recurrence. Simultaneously, integrating emerging technologies such as genomics and proteomics to deeply investigate the molecular characteristics of CEA-elevated MOGCTs could reveal the underlying biological essence and provide clues for developing targeted therapies.

## 5 Conclusions

In summary, this study analyzed clinical data from 123 MOGCT patients and confirmed that tumor stage, metastasis status, and preoperative CEA level are independent risk factors for recurrence after surgical resection of malignant germ cell tumors. Meanwhile, tumor stage and preoperative CEA level are also independent risk factors for postoperative overall survival. Although the routine application of CEA in MOGCTs requires more evidence, this study suggests it may serve as a potential supplementary prognostic indicator. These findings enable clinicians to identify high-risk patients for recurrence and poor prognosis prior to treatment, offering opportunities for early intervention and potentially improving patient outcomes and overall therapeutic efficacy. Additionally, the nomogram we constructed provides clinicians with an intuitive tool for assessing patients’ risks of recurrence and overall survival.

## Data Availability

The raw data supporting the conclusions of this article will be made available by the authors, without undue reservation.
